# Contemporary patients with atrial fibrillation are not anticoagulated despite risks of stroke - Insights from GARDENIA

**DOI:** 10.1371/journal.pone.0354382

**Published:** 2026-07-28

**Authors:** A.K. Kakkar, Marc P. Bonaca, Robert P. Giugliano, Karen Pieper, Dan Bloomfield, Bruce Hug, Janeen Salter, Debra Freedholm, Sanobar Parkar, Saverio Virdone, Herman Sandeep Prakasam, Meg Fluharty, Gloria Kayani, Keith A. A. Fox, on behalf of the GARDENIA investigators

**Affiliations:** 1 Thrombosis Research Institute, London, United Kingdom; 2 CPC Clinical Research/CPC Community Health, Aurora, Colorado, United States of America; 3 TIMI Study Group, Brigham and Women’s Hospital, Harvard Medical School, Boston, Massachusetts, United States of America; 4 Anthos Therapeutics - A Novartis Company, Cambridge, Massachusetts, United States of America; Celal Bayar University: Manisa Celal Bayar Universitesi, TÜRKIYE

## Abstract

**Background:**

GARDENIA is a global, multicentre, prospective, non-interventional study in high-risk atrial fibrillation (AF) patients, who were untreated with anticoagulants.

**Objectives:**

Provide contemporary evidence on reasons for non-treatment with an anticoagulant, and outcomes in high-risk AF patients.

**Methods:**

GARDENIA enrolled 704 AF patients with a CHA_2_DS_2_-VA score ≥2 and an elevated risk of bleeding (older age (≥70 years), reduced renal function, concomitant antiplatelet or NSAID use, or other conditions associated with increased bleeding risk), and had not been recently treated with an oral anticoagulant (OAC). Patients were followed for at least 4 months. The two-year risks of mortality, stroke and major bleeding with or without direct OACs (DOACs) in these patients was estimated using the GARFIELD risk score.

**Results:**

History of major bleeding (18.7%), physician-assessed high-risk of bleeding (17.1%), and patient refusal (17.6%) were the top reasons for not initiating OACs. The 100-person-year event rates (95% CI) for mortality, stroke/systemic embolism (SE)/transient ischemic attack (TIA), and ISTH major bleeding were 13.22 (10.33, 16.92), 2.12 (1.14, 3.94), and 2.21 (1.13, 3.91), respectively. GARFIELD risk analysis indicated a 31% and 40% increase in the risk of mortality and stroke, respectively, and a ~ 30% decrease in risk of major bleeding, compared to the estimated risk, had the patients been anticoagulated. The study was prematurely terminated due to low rate of patient accrual.

**Conclusions:**

Perceived risk of bleeding was the leading cause for non-treatment with OACs in high-risk AF patients. Risk prediction modelling indicated that the benefits of anticoagulation in stroke and mortality reduction outweigh the risk of bleeding.

## Introduction

Atrial fibrillation (AF) is one of the most common heart conditions and a leading cause of mortality and long-term disability, especially in those above the age of 65 [1]. Older patients with AF often live with frailty and other co-morbid conditions that predispose them to an elevated risk of adverse health outcomes, notably stroke [2]. However, despite the high stroke risk, these patients are often under- or untreated with anticoagulants in routine clinical practice [3]. Concerns regarding increased bleeding risk, cognitive impairment, adverse outcomes and polypharmacy are some of the reasons cited for not receiving appropriate anticoagulation [3–5]. Owing to this, observational studies have previously shown that high-risk AF patients often do not receive the net clinical benefit (NCB) from anticoagulation [6], but contemporary evidence is lacking.

Direct oral anticoagulants (DOACs) that selectively target factor IIa (thrombin) or factor Xa avoid many of the limitations of vitamin K antagonists (VKA) and now constitute a majority of anticoagulation prescriptions for AF globally. Nevertheless, the introduction of DOACs has only modestly improved treatment frequency in high-risk patients [7]. A community-based longitudinal study spanning a decade (2011–2021) found that the introduction of DOACs resulted in an increase in the uptake of OACs from 50.2% in 2011 to 59.4% in 2020 [8]. Another study using electronic health record data from the US showed that among patients diagnosed with AF between 2011 and 2021, one in three high-risk patients (36.1%) received no anticoagulation [9]. Gastrointestinal (GI) bleeding remains a clinical concern with DOACs [10,11]. The FRAIL-AF study showed that switching from VKA to DOAC was associated with an increased risk of GI and urogenital bleeding [12]. The increase in thrombotic events among high-risk patients may be linked to adverse downstream consequences of interventions used to manage bleeding, such as interrupting or discontinuing anticoagulation, administering reversal agents and/or prohaemostatic drugs, and transfusing blood products [13].

Consequently, patient registries and observational studies indicate some degree of variability in how clinicians prescribe OACs in patients with AF [14]. The variability is especially high in the high-risk patient population [15]. The consequence of not receiving anticoagulation includes a higher risk of stroke and mortality. A population-based cohort study reported that the 1-year stroke risk doubled with age, from 66 years (0.7%; 95% CI, 0.5%−0.9%) to 74 years (1.7%; 95% CI, 1.3%−2.1%). Despite safety concerns, DOACs appear to have a net clinical benefit of strokes versus bleeding, especially in the older, high-risk AF patients who have comorbidities [16].

The purpose of this registry – A Global prospective observationAl study of Real-worlD managemEnt of patieNts with atrIal fibrillAtion at high risk of stroke (GARDENIA) (NCT05421533) – was to collect practice-based clinical data in a contemporary population of patients with AF with an elevated risk of stroke and yet who are untreated with anticoagulants. The objectives of this registry were to assess the reasons for withholding OACs, the clinical settings in which these patients present, and the associated clinical outcomes [17,18].

## Methods

### Study design and objectives

GARDENIA was a global, multicentre, prospective, observational study that evaluated the baseline characteristics and outcomes of high-risk AF patients who were not on an OAC for stroke prevention. The objectives of the study were to i) evaluate the factors associated with the decision to not treat AF patients with guideline- recommended doses of DOACs for the prevention of stroke and ii) determine the incidence of stroke/systemic embolism (SE)/ transient ischaemic attack (TIA), cardiovascular (CV)- and non-CV related mortality, other major CV events, and health-related quality of life (HRQOL). Enrolment was limited to patients not on OAC at baseline. This included patients whose risk factors indicated a need for OAC, yet who were not on an anticoagulation regimen at the time of enrolment due to various concerns. The study enrolled 704 patients across 12 countries from September 2022 to June 2024, who were followed up for a minimum of 4 months from the time of enrolment.

### Study population

Full inclusion and exclusion criteria for the study population are detailed in S1 Table. The study enrolled patients diagnosed with AF or atrial flutter (documented by an ECG or cardiac monitor), who had a CHA_2_DS_2_-VA score of 2 or more (excluding female sex as a risk factor) and were not receiving OACs at the time of enrolment. In addition, the patients met one or more of the following conditions: (i) age 70 years or older; (ii) reduced renal function (creatinine clearance (CrCl) <30 mL/min); (iii) chronic use of non-steroidal anti-inflammatory drugs (NSAIDs) or antiplatelet agents; and (iv) any other condition associated with increased risk such as history of major or clinically-relevant non-major (CRNM) bleeding, risk of fall or frailty. Patients who had, or would require, a mechanical heart valve, or who had a left atrial appendage occlusion device at the time of screening, were excluded from the study (S1 Table). Frailty was assessed based on the physician’s input in the electronic case report form (eCRF). The physician or site staff responded either yes or no to the question, ‘Do you assess the patient to be frail?’.

### Enrolment protocol

Potential study participants were identified during routine clinical care or through medical chart review. Medical data review to identify potential participants included AF diagnosis, age, renal function, relevant bleeding history, chronic use of NSAID medications (yes/no), use of antiplatelet medication(s) (yes/no), indication for antiplatelet use, and use of an OAC (yes/no). A pre-screening log containing the above non-identifying information was used to document screening efforts, enrolment into the registry, or reasons patients did not enrol (e.g., screen failure, refusal). Potential patients, or their legally authorised representative, were contacted about the study during routine clinical care or by telephone or email. Patients interested in enrolling in the study were scheduled for a screening/baseline visit, at which point informed consent was obtained.

### Study close-out

The GARDENIA study was originally intended to enrol 1500 OAC-untreated high-risk AF patients and to have a 24-month follow-up period. The study was prematurely terminated 24 months after commencement due to the challenges of recruiting patients within the planned study milestones. Enrolment was closed, and follow-up for at least 4 months was allowed for all patients to ensure sufficient data collection to characterise the study population.

### Assessment of risk using the GARFIELD risk score

The GARFIELD risk score has been previously described [19]. It was derived and validated based on data from 52,080 newly diagnosed AF patients and their 2-year clinical outcomes [20]. This tool allows one to calculate the likelihood of all-cause mortality, of a non-haemorrhagic stroke, and of a major bleed. It provides predicted risk for each day from initial diagnosis to two years and gives trajectories of risk assuming one is using VKA, a DOAC, and no oral anticoagulant. In other words, one has expected risk for each of the three outcomes, assuming each of the three possible treatment decisions, across a two-year period.

### Statistical design and analysis

Categorical variables are described by frequencies and percentages, and continuous variables by medians and 25th and 75^th^ percentiles. Renal function was calculated using CrCl from the Cockcroft-Gault estimation [21].

Rates of mortality, CV outcomes, bleeding, and subsequent OAC use were measured as numbers of events and Kaplan-Meier (K-M) rates. Because all patients were to receive a 4-month follow-up, rates are reported at that time. Events were measured from the time of enrolment in the study until the first occurrence of the event of interest, end of follow-up, or 4 months, whichever occurred first.. Several patients were in the study longer than 4 months. Therefore, the K-M event rates were also presented at 8 months. All events occurring after the last time point of interest were censored. The 100-person-year event rates were also calculated for the end-of-observation period. The CHA_2_DS_2_-VA score was calculated using eCRF data on Congestive heart failure, Hypertension, Age (2 points for age ≥ 75), history of Diabetes, history of Stroke/SE/TIA (2 points) and Vascular disease (CHA_2_DS_2_-VA) [22]. A modified HAS-BLED score, excluding labile INR was calculated using eCRF data on hypertension, age, stroke history, prior bleeding, history of renal and liver disease, and history of alcohol use [23]. Labile INR was not included, as the patients were not on warfarin treatment.

In the subset of patients who were given an OAC during the follow-up period, the time from enrolment to initiation of OAC use is displayed using a cumulative incidence curve. The EQ-5D-5L questionnaire was completed by the patients at baseline and at the 4-month visit.

Estimated event rates to 2 years (all-cause mortality, non-haemorrhagic stroke, major bleed) were calculated for all patients for each of two potential treatment decisions (DOAC, no OAC) by applying the GARFIELD risk scores [19]. The average of each of these event rates across time for each treatment arm illustrated the expected outcomes had this set of patients been followed for two years and had they received the treatment of interest. Overlaid was the K-M mortality curve from the GARDENIA population as a measure of calibration. There were not enough stroke or bleeding events in the study to provide similar curves.

### Ethical approval

Independent ethics committee and hospital-based institutional review board approvals were obtained, as necessary, for the registry protocol. Additional approvals were obtained from individual study sites. GARDENIA was conducted in accordance with the principles of the Declaration of Helsinki, local regulatory requirements, and the International Conference on Harmonisation Good Pharmacoepidemiological and Clinical Practice Guidelines. Written informed consent was obtained from all study participants.

### Inclusivity in global research

Additional information regarding the ethical, cultural, and scientific considerations specific to inclusivity in global research is included in the Supporting Information

## Results

### Baseline characteristics

The baseline characteristics of the patients enrolled in the study are shown in Table 1. The study screened 765 patients between September 2022 and June 2024; those who did not meet the inclusion criteria or who did not consent were excluded. A total of 704 (92% of those screened) participants across 12 countries were enrolled. The top three countries by enrolment were Argentina (153; 21.7%), Poland (109; 15.5%), and the United Kingdom (99; 14.1%). The median age was 79 (Q1, Q3: 73, 86) years, and 41.2% of the participants enrolled were female; 398 (58.7%) participants were determined to be frail by the treating physician. The median (Q1, Q3) CHA_2_DS_2_-VA score was 4 (3, 5). The majority of the patients enrolled (92.5%) had prevalent AF. Of the 704 patients enrolled, end-of-study information was available for 697 patients. There were 542/697 (77%) and 311/697 (44%) patients in the study at the 4- and 8-month time points, respectively (S10 Table).

**Table 1:** Distribution of baseline characteristics.

Baseline characteristics	GARDENIA
	(N = 704)
Country, n (%)	
Argentina	153 (21.7)
Poland	109 (15.5)
United Kingdom	99 (14.1)
Czech Republic	73 (10.4)
United States of America	66 (9.4)
Germany	46 (6.5)
Mexico	44 (6.3)
Brazil	39 (5.5)
Spain	26 (3.7)
Italy	25 (3.6)
Canada	20 (2.8)
Hungary	4 (0.6)
Sex, n (%)	
Male	414 (58.8)
Female	290 (41.2)
Age, median (Q1, Q3), years	79.0 (73.0, 86.0)
Race/Ethnicity, n (%)	
White	601 (91.1)
Asian	9 (1.4)
Black or Afro-Caribbean	9 (1.4)
Other	41 (6.2)
Unknown	43
Vital signs	
Frail according to investigator*	398 (58.7)
Height (Q1, Q3) (cm)	167.0 (160.0, 174.0)
Weight (Q1, Q3) (kg)	75.3 (65.0, 86.0)
Body mass index, median (Q1, Q3), kg/m²	27.0 (24.0, 30.4)
Systolic blood pressure, median (Q1, Q3), mmHg	129 (117, 140)
Diastolic blood pressure, median (Q1, Q3), mmHg	73 (67, 80)
Pulse, median (Q1, Q3), bpm	70 (62, 80)
Type of atrial fibrillation, n (%)	
Paroxysmal	304 (44.9)
Permanent	286 (42.2)
Persistent	72 (10.6)
Not Yet Determined	15 (2.2)
Symptomatic AF, n (%)	269 (40.1)
AF Timing, n (%)	
Incident	53 (7.5)
Prevalent	650 (92.5)
Care setting	
Hospital – Private	128 (18.2)
Hospital – Public	241 (34.2)
Office – Group	295 (41.9)
Office – Solo	40 (5.7)
Care setting specialty at diagnosis, n (%)	
Cardiology	482 (68.5)
Primary care/general practice	112 (15.9)
Internal medicine	99 (14.1)
Geriatrics	5 (0.7)
Neurology	1 (0.1)
Care setting location at diagnosis, n (%)	
Office	405 (57.5)
Hospital	265 (37.6)
Anticoagulation clinic/thrombosis centre	12 (1.7)
Emergency room	4 (0.6)
Unknown	18 (2.6)
Medical history, n (%)	
Hypertension	604 (86.3)
Hypercholesterolaemia	324 (47.4)
Prior bleeding	283 (40.5)
Prior GI Bleed	139 (20.2); (49.1% of 283)
Prior intracranial bleed	43 (6.2); (15.2% of 283)
Heart failure	259 (37.1)
NYHA Class	
I	28/239 (11.7)
II	156/239 (65.3)
III	53/239 (22.2)
IV	2/239 (0.8)
Diabetes	196 (28.1)
Coronary artery disease	189 (28.7)
Hypothyroidism	111 (16.0)
Clinically significant valve disease	100 (14.5)
Aortic stenosis	35 (5.1)
Aortic regurgitation	24 (3.5)
Mitral stenosis	10 (1.4)
Mitral regurgitation	50 (7.2)
Tricuspid regurgitation	43 (6.2)
Prior stroke/ TIA/ SE	112 (16.2)
Prior stroke	77 (11.0)
Prior transient ischaemic attack	37 (5.3)
Prior systemic embolism	11 (1.6)
Prior MI	93 (13.4)
Peripheral artery disease	80 (11.7)
Aortic disease	77 (11.4)
Cancer	76 (10.9)
Dilated cardiomyopathy	58 (8.4)
Dementia/ or Cognitive Impairment	49 (7.0)
Carotid disease	44 (6.7)
Sleep Apnoea	34 (5.1)
Prior VTE	26 (3.8)
Cirrhosis	16 (2.4)
Hyperthyroidism	10 (1.4)
Congenital cardiac anomaly	7 (1.0)
Rheumatic heart disease	7 (1.0)
Intracardiac thrombus	4 (0.6)
Alcohol consumption, n (%)	
Abstinent	482 (73.6)
Light (<8 drinks per week)	146 (22.3)
Moderate (8–13 drinks per week)	19 (2.9)
Heavy (≥14 drinks per week)	8 (1.2)
Smoking status, n (%)	
Non-smoker	445 (66.0)
Ex-smoker	184 (27.3)
Current smoker	45 (6.7)
Creatinine Clearance (Cockcroft-Gault), median (Q1, Q3)	48.8 (33.1, 71.8)
Creatinine Clearance Groups, n (%)	
< 15	30 (7.1)
15-30	65 (15.3)
31-50	122 (28.7)
> 50	208 (48.9)
CHA_2_DS_2_-VA score, median (Q1, Q3)	4 (3, 5)
0-2	52 (8.0)
3	144 (22.3)
4	183 (28.3)
5	143 (22.1)
>=6	124 (19.2)
HAS-BLED score, median (Q1, Q3)	2 (3, 3)
0-1	10 (1.6)
2	170 (26.6)
3	302 (47.3)
>=4	157 (24.6)
EQ-5D-5L scores (N and % with slight to extreme symptoms)	
Mobility	436/671 (65.0)
Self-Care	242/671 (37.0)
Activity Level	394/671 (58.7)
Pain	439/671 (65.4)
Anxiety/ Depression	315/671 (46.9)

Notes:

*Frailty was a question in the electronic case report form, and the treating physician answered (Yes/No) based on their assessment.

Denominators are provided when percentages are based on subgroup totals.

AF Timing is derived as time difference (days) between date of AF diagnosis and enrolment date. If the difference is ≤ 24 days, then incident. If the difference is > 24 days, then prevalent. AF Timing is missing on 60 patients.

Creatinine Clearance is missing in 279 patients; HAS-BLED, 103; alcohol consumption, 49; carotid artery disease has, 49; coronary artery disease, 46; race, 44; CHA2DS2-VA score 58;

All others had less than 5% missing.

Baseline EQ-5D-5L data were available for 671 out of the 704 patients enrolled in the study.

The median days from creatinine lab measure to enrolment was 37 days (Q1, Q3: 5, 140).

The medical history and care setting where the patients were enrolled are detailed in Table 1. Prior stroke/SE/TIA and history of bleeding were recorded in 112 (16.2%) and 283 (40.5%) patients, respectively. The majority of participants in the study, 482 (68.5%), were identified by cardiologists at clinician office sites. The EQ-5D-5L patient-reported outcome at baseline indicated that >50% of the enrolled patients had an observable limitation with mobility, activity level, or experienced some form of pain. Slight to extreme anxiety/depression and limitations to self-care were reported by 46.9% and 37% of the patients, respectively.

### Reasons for withholding oral anticoagulants

Table 2 shows the reasons OACs were withheld throughout the duration of the study. The table depicts both the primary reason (n = 638) and the primary plus the additional reasons (n = 1170) for withholding OACs. Concerns about bleeding risk were the main reason for not initiating an OAC. A previous history of bleeding needing hospitalisation was stated by 119 (18.7%), while 109 patients indicated an underlying condition that was associated with bleeding. Patient refusal to take an OAC was noted in 112 (17.6%) of those enrolled. The physician’s decision to withhold anticoagulants was the most common reason for not being treated with an OAC (n = 261, 47.3%), followed by a combined decision between the physician and the patient (n = 186, 33.7%) (S2 Table).

**Table 2:** Distribution of main reason anticoagulant was not used (n = 638 who responded).

Main reason anticoagulant was not used	Primary Reason* (N = 638)	Any: primary and additional reasons** (N = 1170)
Previous bleeding needing hospitalization or medical intervention	119 (18.7)	153 (13.1)
Patient refusal to take anticoagulants	112 (17.6)	149 (12.7)
Underlying condition associated with bleeding risk	109 (17.1)	173 (14.8)
Fall risk/ History of traumatic falls	39 (6.1)	108 (9.2)
Frailty	35 (5.5)	127 (10.9)
Previous minor or nuisance bleeding	33 (5.2)	63 (5.4)
Predominantly in sinus rhythm/ Low AF burden	27 (4.2)	41 (3.5)
Severe renal impairment	18 (2.8)	42 (3.6)
Already taking anti-platelet drugs for other medical condition	17 (2.7)	65 (5.6)
Chronic NSAID use (>3 times per week)	14 (2.2)	55 (4.7)
Drug interactions	6 (0.9)	7 (0.6)
Haemodialysis	5 (0.8)	16 (1.4)
Anticoagulant compliance concern/ Poor access to monitoring	2 (0.3)	11 (0.9)
Cost	NA	11 (0.9)
Cognitive Impairment	NA	8 (0.7)
Liver disease	2 (0.3)	6 (0.5)
Alcohol abuse	2 (0.3)	2 (0.2)
Other	43 (6.7)	43 (3.7)
Unknown	55 (8.6)	90 (7.7)

*66 patients had Reason Not Given missing

** Not mutually exclusive responses. Some patients had up to 7 answers given.

A subset of 59 participants who were not on an OAC at the time of enrolment were anticoagulated shortly after study initiation. S1 Fig shows the timing of prescription of OACs to one year. Fifty-seven patients with treatment timing data went on to receive an OAC, and 49 of these were during the initial 4-month period [median time to start of OAC: 42 days (Q1, Q3: 11, 73)]. The breakdown of patients is shown in S3 Table. S4 Table provides the reason given for initiating OAC treatment. Three patients were recorded as having experienced stroke/TIA and 7 patients were no longer deemed to be at high bleeding risk.

**Fig 1:**
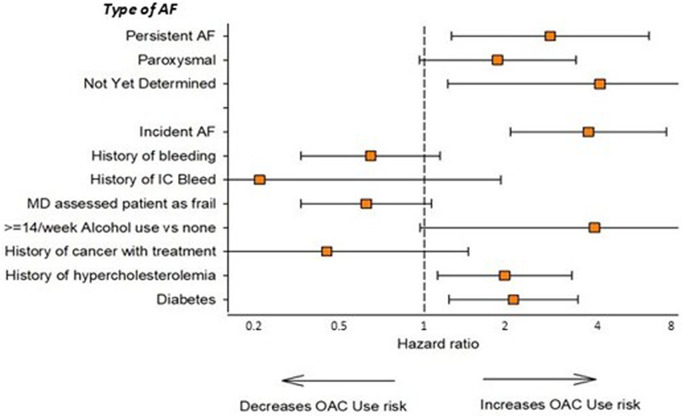
Factors most associated with the decision to start an OAC (reference for type of AF is Permanent).

Fig 1 shows the key factors associated with OAC use. Type of AF, history of hypercholesterolemia, and diabetes were associated with an increase in OAC usage. Frailty was associated with a decrease in OAC use. The complete list of baseline characteristics of patients who started OAC after enrolment in the study are provided in S5 Table.

### AF treatment strategy

S6 Table presents the other AF management strategies by baseline OAC status. Prior cardioversion procedures were reported in 76 (12.1%) and 9 (15.3%) participants, and a history of ablation was recorded in 47 (7.4%) and 3 (5.1%) participants who did not start on OACs, and who started on OACs, respectively. None of the patients enrolled had a prior left atrial appendage procedure, as this was an exclusion criterion for the study.

S7 Table shows the baseline medications at the time of enrolment. Notably, more than 85% of the patients enrolled were taking some form of CV medications, and 56.5% were on beta blockers.

### Clinical outcomes

The event rates at 4-months, 8-months and for 100-person years for the selected clinical outcomes for 697 patients with follow-up data are shown in Table 3. A total of 44 and 53 deaths were recorded during the 4-month period and the 8-month period, respectively. The 4-month, 8-month and 100-person year mortality rates (95% CI) were 6.48 (4.86, 8.62), 8.53 (6.54, 11.08) and 13.22 (10.33, 16.92), respectively. The CV deaths primarily occurred in the first four months.

**Table 3:** Kaplan-Meier event rates at each time point and per 100 person-years for the end of observation for selected outcomes by time since AF diagnosis. n = 697.

	K-M 4-month		K-M 8-month		Per 100-Person-years	
Outcome	Events	Rate (95% CI)	Events	Rate (95% CI)	Events	Rate (95% CI)
All-Cause mortality	44	6.48 (4.86, 8.62)	53	8.53 (6.54, 11.08)	63	13.22 (10.33, 16.92)
Cardiovascular	12	1.83 (1.04, 3.20)	14	2.25 (1.33, 3.80)	14	2.94 (1.74, 4.96)
Non-Cardiovascular	21	3.14 (2.06, 4.78)	24	3.80 (2.55, 5.64)	31	6.51 (4.58, 9.25)
Stroke	5	0.74 (0.31, 1.77)	6	0.95 (0.42, 2.13)	8	1.69 (0.85, 3.38)
Ischemic	4	0.68 (0.22, 1.55)	5	0.80 (0.33, 1.93)	7	1.48 (0.71, 3.1)
Haemorrhagic	0	–	0	–	0	–
Unknown Type	1	0.15 (0.02, 1.09)	1	0.15 (0.02, 1.09)	1	0.21 (0.03, 1.49)
TIA	1	0.16 (0.02, 1.10)	2	0.40 (0.10, 1.66)	2	0.42 (0.11, 1.68)
Stroke/TIA	6	0.89 (0.40, 1.98)	8	1.35 (0.67, 2.73)	10	2.12 (1.14, 3.94)
Stroke/ SE	5	0.74 (0.31, 1.77)	7	1,20 (0.56, 2.54)	9	1.90 (0.99, 3.66)
Stroke/ TIA/ SE	6	0.89 (0.40, 1.98)	8	1.35 (0.67, 2.73)	10	2.12 (1.14, 3.94)
SE	0	–	1	0.25 (0.03, 1.74)	1	0.21 (0.03, 1.49)
Venous Thromboembolism	4	0.60 (0.23, 1.60)	4	0.60 (0.23, 1.60)	4	0.84 (0.32, 2.24)
Heart Failure	25	3.81 (2.59, 5.59)	32	5.38 (3.81, 7.57)	36	7.78 (5.61, 10.79)
Myocardial Infarction	7	1.08 (0.52, 2.26)	7	1.08 (0.52, 2.26)	7	1.47 (0.70, 3.09)
Cardioversion	7	1.07 (0.51, 2.24)	8	1.36 (0.67, 2.77)	10	2.14 (1.15, 3.97)
Hospitalization	71	10.77 (8.63, 13.40)	80	12.73 (10.33, 15.65)	91	20.93 (17.04, 25.71)

7 patients had no end of study information.

The causes of death at 4 months and 8 months are shown in S8 Table. It is worth noting that 21/44 deaths at 4 months were due to non-CV reasons. Among the CV causes of death, six out of the 12 deaths were due to congestive heart failure. At both the 4-month and 8-month timepoints, 11 and 15 deaths were due to unknown reasons, respectively.

The rates of the composite stroke/TIA/SE at 4-months, 8-months, and per 100-person years based on the full observation period were 0.89 (0.40, 1.98), 1.35 (0.67,2.73) and 2.12 (0.03, 1.49), respectively. The one SE event at 8-months timepoint was recorded after the patient had a primary event of stroke, hence did not contribute to the composite stroke/TIA/SE per 100-person event rate. The rates of heart failure at the three time periods were 3.81 (2.59, 5.59), 5.38 (3.81, 7.57) and 7.78 (5.61, 10.79) (Table 3).

The K-M event rates for ISTH major bleeding at 4-months and per 100-person years based on full observation period are shown in Table 4. It is notable that all the major and CRNM bleeding events occurred within the first four months,. One death due to intracranial/spinal haemorrhage was recorded at the 4-month period (S8 Table). The bleeding rates for the 697 patients while not on an OAC are shown in S9 Table. Of the 59 patients who started an OAC, only one had a bleed event subsequent to OAC initiation. The patient had a major upper gastrointestinal bleed event that was captured at the 4-month timepoint.

**Table 4:** ISTH bleeding Kaplan-Meier event rates at the 4-month timepoint and per 100 person-years for the end of observation.

	K-M 4-month		Per 100 Person years	
Outcome	Events	Rate (95% CI)	Events	Rate (95% CI)
Major	10	1.52 (0.82, 2.81)	10	2.21 (1.13, 3.91)
Intracranial	1	0.16 (0.02, 1.11)	1	0.21 (0.03, 1.49)
Non-IC Major	9	1.36 (0.71, 2.60	9	0.19 (0.01, 0.36)
CRNM	2	0.29 (0.07, 1.17)	2	0.42 (0.11, 1.68)
Minor	11	1.70 (0.94, 3.05)	11	2.34 (1.29, 4.22)
Major or CRNM	12	1.81 (1.03, 3.17)	12	2.53 (1.44, 4.46)
Any	23	3.50 (2.34, 5.23)	23	4.92 (3.27, 7.40)

Note: One of the Major bleeds occurred after a patient started OAC.

*Major bleeding including all major bleeding + intracranial bleeding

The per-100-person-year rates of clinical outcomes based on the primary reason for not receiving anticoagulants are listed in S10 Table. Due to the low number of events, the rates presented must be viewed with caution.

The patient disposition at 4-, 8-month and end of study are shown in S11 Table. The reason for withdrawal from the study are shown in S12 Table.

### Assessment of risk using the GARFIELD risk tool

**Fig 2:**
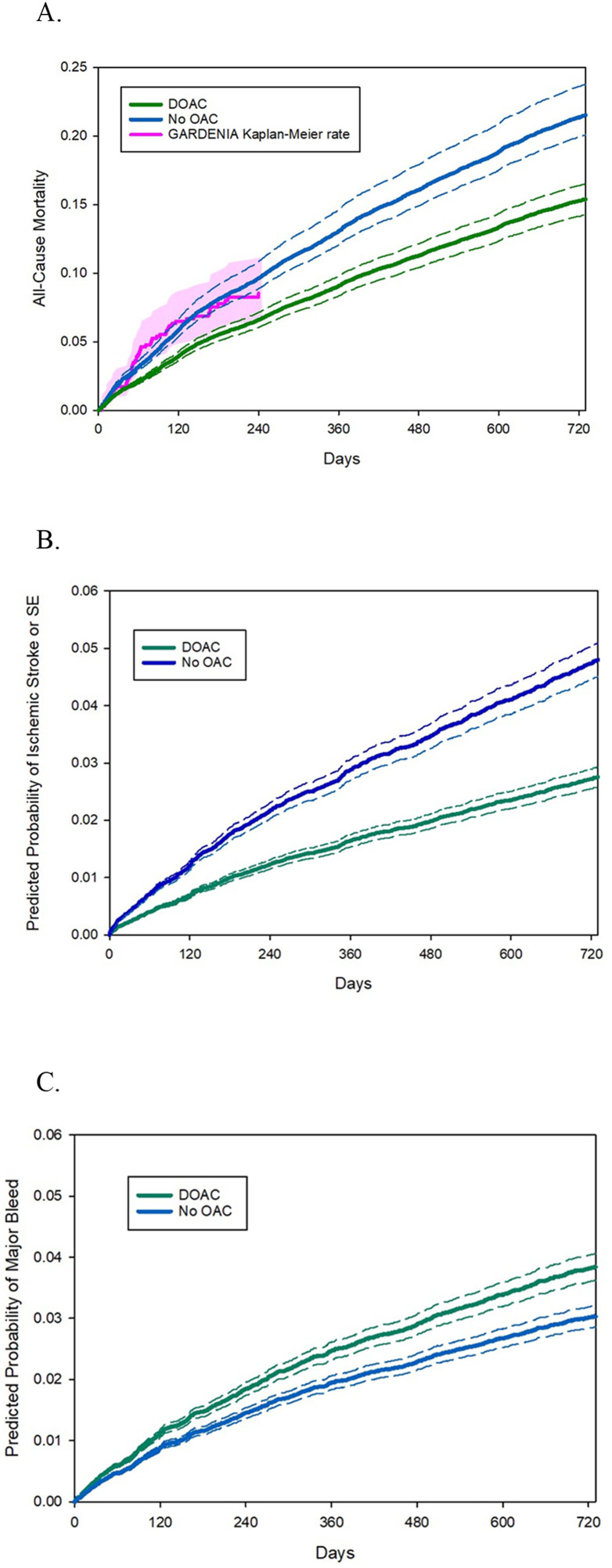
GARFIELD- Risk score assessment.

The actual mortality rate closely aligned with the estimated mortality rate assuming no OAC treatment using the GARFIELD risk tool (Fig 2a). Both mortality and non-haemorrhagic stroke were estimated to be significantly reduced over the two-year period with the use of a DOAC instead of no OAC in this cohort (Figs 2a and 2b). Also expected, major bleeding rates with DOAC were estimated to be higher than with no OAC use (Fig 2c). Observed rates for ischaemic stroke and bleeding are not presented as there were only 4 strokes and 10 major bleeds at the 4-month timepoint.

The expected 2-year risk of non-haemorrhagic stroke/SE with no OAC (4.8%), decreased by over 40% to 2.8% risk with a DOAC in these same patients (Fig 2b). The estimated difference in major bleeding risk was 3.8% in the DOAC groups vs 3.0% in the no-OAC group (Fig 2c). The covariates used in the risk calculator, and their comparative baseline characteristics between the GARDENIA and GARFIELD-AF populations, are shown in S13 Table.

S14 Table shows the estimated 2-year rates of non-haemorrhagic stroke/SE and major bleeding in patients who remained untreated with an OAC through the duration of the study, and those who were initiated on OAC treatment. The rates were calculated based on the GARFIELD risk rates, assuming the patients were not given an OAC for stroke prevention. The 2-year rates for patients with an OAC treatment were slightly lower than those who were OAC untreated, but the confidence intervals were wide and overlapped between the two groups.

## Discussion

The GARDENIA study enrolled AF patients who are at high-risk of stroke, and yet are not on an OAC regimen. Hypertension was the most common comorbidity (86.3%), while approximately 40% had prior history of bleeding, and 58.7% were deemed frail by the treating physician. The top reasons for not receiving an OAC were an underlying condition that was associated with bleeding risk, or prior history of bleeding. The decision to not initiate an OAC was either taken by the treating physician or was a joint decision between the physician and the patient. The 100-person-year event rates for all-cause mortality, composite of stroke/TIA/SE, and ISTH major bleeding were 13.22, 2.12 and 2.21, respectively. These rates were consistent with the GARFIELD mortality risk for patients not on a DOAC. With respect to mortality, based on the baseline characteristics of the GARDENIA patients, the average one-year predicted mortality rate when untreated with an OAC would be 13.2% (12.2%, 14.8%) using the GARFIELD mortality risk score. Taken together, this contemporary study highlights the sizable population of OAC-untreated, high-risk AF population, who are not receiving the net clinical benefit from being anticoagulated.

It is well established that high-risk AF patients are consistently inappropriately anticoagulated, due to safety concerns and perceived bleeding risk [24,25]. A 2020 analysis of Medicare data indicated that 67.1% of AF patients did not receive any OAC within the first year of AF diagnosis, with older age, dementia, frailty, anaemia, and a history of pelvic or hip fracture among predictors of non-initiation of OACs [26]. Similar results were found from meta-analyses reports from New Zealand, South Korea, Sweden and the United Kingdom [5,27]. The findings from this study align with the established evidence regarding not receiving OACs and underscore a need for a contemporary update to the status of non-anticoagulated AF patients and their clinical outcomes

Risk of bleeding, especially gastrointestinal bleeding, continues to be a major consideration when deciding on an OAC treatment strategy [28,29]. The risk of bleeding is further compounded in older and frail AF patients, and in those with renal dysfunction and other risk factors such as diabetes and hypertension. The GARDENIA study indicated that, in 81% of cases, the reason for not receiving an OAC was based on the physician’s decision or a joint decision between the patient and physician. Furthermore, while non-significant, physician-assessed frailty, history of bleeding, and history of cancer treatment were the strongest predictors of non-treatment with OAC. This highlights a larger perceived risk of bleeding by the treating physician and patient, and an elevated risk aversion behaviour. A similar outcome was also seen in the untreated cohort of the PINNACLE registry in the US. After re-review, 27.1% of the physicians would go on to reconsider prescribing OACs [30].

In GARDENIA, ~ 10% of the physicians changed their decision and prescribed OAC after the study began. Further, among patients who received OAC after enrolment, 22.6% had incident AF. This indicates that the study may have served as a platform for identifying high-risk OAC-naïve AF patients who eventually benefited from anticoagulation, a trend which has been reported in other studies [31]. The ESC and ACC/AHA/ACCP/HRS guidelines for AF management recommend periodic re-evaluation of newly diagnosed AF patients to determine the evolving risk of thromboembolism [1,32]. The result from GARDENIA provides further evidence thatperiodical re-evaluation of the anticoagualtion strategy of high-risk AF patients is required.

The 100-person year all-cause mortality rate seen in this study (13.22) was higher than the 2-year outcomes seen in GARFIELD-AF (3.79) registry and the two-year outcomes of ORBIT-AF registry (5.46 in women and 5.74 in men per 100-patient years, respectively) [33,34]. However, the proportion of OAC-untreated patients in these studies was lower than in GARDENIA. In GARDENIA ~ 91% (640/697) of the participants were untreated with an OAC for the duration of the study, and 58.7% of the participants were considered to be frail by the treating physician. The mean age and median CHA_2_DS_2_-VAsc score were 79 and 4, respectively. The population in the ELDERCARE-AF study is a closer comparator to this study. In the placebo arm of the ELDERCARE-AF study, the mean age and CHA_2_DS_2_-VASc score were 86.4 and 5.0, respectively. The all-cause mortality 100-person year rate in this arm was 10.2, which trends closer to that seen in the GARDENIA study [25].

With respect to the stroke rates, the 100-person-year event rate for stroke/SE estimated in GARDENIA at 2.12 was lower than the placebo arm of ELDERCARE-AF. We reason that the estimated stroke rate is lower due to limited enrolment and the short duration of follow-up (4-months). There were 6 stroke/SE/TIA events in the first 4-months of the study, and ~9% of the patients had started on OAC treatment after enrolment. This is likely to reduce the expected rate of stroke.

Finally, the estimated ISTH major bleeding 100-person-year rates observed in the OAC-untreated patients in this study (2.21%) were comparable to those in the placebo arm of the ELDERCARE-AF study (1.8%).

To predict outcomes in these patients, we used the GARFIELD risk score. While other mortality, stroke and bleeding risk predictors are available, we chose the GARFIELD risk score as it allowed calculation of risk for each day of the 2-year period, by treatment, rather than providing a single integer per patient [19]. The analysis of these patients indicated that they indeed corroborate with the predicted risk of mortality, stroke and bleeding in the OAC untreated patient population and the risk is reduced if they are assumed to have initiated a DOAC regimen. Further investigation is warranted, with a longer follow-up period to better understand the clinical outcomes in this population. Nevertheless, we believe clinicians would benefit from having access to such risk prediction tools that help them determine the evidence-based risk of outcomes in their patients.

### Strengths and limitations

This is the first international registry conducted in the DOAC era that focussed on high-risk AF patients who were OAC naïve at the time of enrolment. Epidemiological studies and analyses of claims databases have suggested that a substantial proportion of older and high-risk AF patients do not receive appropriate OAC treatment, and hence are not attaining the net clinical benefit of anticoagulation. This study furthered our understanding of the characteristics of the OAC untreated population, studied the reasons why they remain untreated, and highlighted the resource burden they impose on the healthcare system, due to the high hospitalisation rate that was recorded. Finally, the GARFIELD risk assessment showed that the predicted stroke and mortality rate in these patients would have been lower had they received DOACs.

This study has limitations: since only OAC untreated patients were considered, the patients were not consecutively enrolled; therefore, it is not a comprehensive reflection of the patient population presenting at the clinic. Further, due to low enrolment, detailed statistical modelling regarding clinical outcomes in association with the treatment status was not performed. In addition, because the study follow-up was truncated to 4-months, long-term follow-up of the participants was not conducted. There were limited number of patients to determine the event rate at one year and to allow a closer comparison with the event rates from other studies which had a longer follow-up. The challenges in enrolment also signalled that while the untreated population existed, there were difficulties in accessing these patients for registries and clinical trials. Closer evaluation of strategies to enrol untreated AF patients must be conducted to improve recruitment and retention in future studies. Finally, the GAFIELD risk analysis was a post-hoc decision.

## Conclusion

The GARDENIA registry was designed to provide a contemporary understanding of the characteristics and treatment of patients who are OAC untreated. The study demonstrated the urgent need to further evaluate this population, who can receive net clinical benefit from anticoagulation. Further, the use of an individualised risk score to predict outcomes provides a precedent for future studies and clinical practice. Finally, the study highlights a clear unmet need for safer anticoagulants with reduced bleeding risk. Factor XI inhibitors have consistently shown a safer bleeding profile [35]. However, their efficacy in stroke prevention warrants further evidence. The patient population studied in this registry may represent a key subset that could benefit from these emerging anticoagulants once stronger evidence of stroke-prevention efficacy is available.

### Key learning points

What is already known:

- Older AF patients and those with elevated stroke risk factors are prone to under-treatment or being untreated with anticoagulants in routine clinical practice.
- Concerns about bleeding risk, cognitive impairment, and adverse reactions from polypharmacy are the main reasons for withholding oral anticoagulants (OACs), leaving high-risk AF patients without the net clinical benefit of anticoagulation.
- The introduction of DOACs has contributed to a marginal improvement in treatment frequency among high-risk AF patients, but a substantial proportion of patients remain untreated.

What this study adds:

- The Global prospective observationAl study of Real-worlD managemEnt of patieNts with atrIal fibrillAtion at high risk of stroke (GARDENIA) study is the first international registry conducted in the DOAC era that focused on high-risk AF patients who were OAC naïve at the time of enrolment.
- The registry highlights the contemporary reasons why many high-risk AF patients remain untreated with OACs. An underlying condition associated with bleeding risk remains the top reason for not initiating OAC treatment.
- Assessment of the registry data using the GARFIELD risk prediction tool showed that the predicted stroke and mortality rates in these patients would have been lower had they received DOACs.

## Supporting information

S1 Data GARDENIA supplementary materials - 26Jun2026. (DOCX)

S1 Table Inclusion and exclusion criteria. (DOCX)

S2 Table The person who decided that OAC would not be used. (DOCX)

S3 Table OAC uptake by patients after enrolment. (DOCX)

S4 Table Reason given for starting OAC treatment. (DOCX)

S5 Table Baseline characteristics associated with those who started OAC. (DOCX)

S6 Table Distribution of baseline characteristics in GARDENIA: Atrial Fibrillation Strategy. (DOCX)

S7 Table Baseline Medications. (DOCX)

S8 Table Cause of Death. (DOCX)

S9 Table ISTH Bleeding rates while not on an OAC. Patients who start an OAC are censored at the time of the OAC.
(DOCX)

S10 Table Clinical events based on main reason for not being treated with anticoagulants (per 100-person-year rates for end of observation time). (DOCX)

S11 Table Disposition status at different time points during follow-up (697 with end of study information). (DOCX)

S12 Table Reasons for withdrawal. (DOCX)

S13 Table Comparing the GARDENIA patients to the GARFIELD-AF patients with CHA2DS2-VA of 2 or greater who did not receive any OAC as their initial treatment. GARFIELD-AF is patients with newly diagnosed AF.
(DOCX)

S14 Table GARFIELD estimated rates of outcomes. (DOCX)

S1 Fig Cumulative incidence of OAC uptake in those patients who went on to take an OAC during the follow-up period. Note: Of the 697 patients with follow-up information, 57 were subsequently started on an OAC. (59 patients have an observation of OAC use but two do not have a start date for the OAC so we cannot confirm if it was before or after the start of enrolment). The median time to the start of an OAC was 42 days (11, 73).
(JPG)

## Abbreviations

AF: Atrial fibrillation
CrCl: Creatinine clearance
CRNM: Clinically relevant non-major bleeding
CV: Cardiovascular
DOAC: Direct oral anticoagulant
eCRF: Electronic case report form
HRQOL: Health-related quality of life
K-M: Kaplan-Meier
MI: Myocardial infarction
INR: International Normalised Ratio
NSAID: Non-steroidal anti-inflammatory drugs
OAC: Oral anticoagulants
TIA: Transient ischaemic attack
VTE: Venous thromboembolism
