# Supplementary material for: Contemporary patients with atrial fibrillation are not anticoagulated despite risks of stroke - Insights from GARDENIA
Source: PLoS One. 2026 Jul 28;21(7):e0354382. doi: 10.1371/journal.pone.0354382 (PMC13411893; doi:10.1371/journal.pone.0354382)
Supplement: S1 Data — (DOCX) [file pone.0354382.s001.docx]

**Contemporary patients with atrial fibrillation are not anticoagulated despite risks of stroke - Insights from GARDENIA**

**Supplementary material**

**Table S1. Inclusion and exclusion criteria**

| Inclusion criteria | 1. Written informed consent from the patient, or legally authorized representative, must be obtained before any assessment is performed 2. Patients with diagnosed AF or atrial flutter (documented on an electrocardiogram or monitor recording) 3. CHA₂DS₂-VASc score ≥2 (excluding female as a factor) 4. Judged by the investigator to be at increased risk of bleeding because of at least 1 of the following:    1. Age ≥70 on the day of informed consent    2. Reduced renal function (creatinine clearance <30 mL/min by Cockcroft-Gault)    3. Chronic use of NSAIDs or antiplatelet agents    4. Any other condition associated with increased risk such as a prior history of major or clinically relevant nonmajor bleeding, increased fall risk, or frailty. 5. Patients who are not treated with oral anticoagulants (only applies to Phase 1) |
| --- | --- |
| Exclusion criteria | 1. Mechanical heart valve or valve disease that is expected to require valve replacement intervention (surgical or invasive) during the course of the registry study 2. AF due to a reversible cause (e.g., cardiac surgery, pulmonary embolism, untreated hyperthyroidism, ethanol use) 3. Clinically unstable or active endocarditis or endovascular infection 4. Patients with a medical condition other than AF for which chronic use of an oral anticoagulant is indicated 5. History of left atrial appendage closure or removal 6. Life expectancy <1 year at the time of enrollment as assessed by the Investigator 7. Any medical or psychiatric condition which in the judgment of the Investigator may preclude patients of complying with study requirements for the duration of the study |

**Table S2. The person who decided that OAC would not be used.**

| **Deciding party** | **N=552** |
| --- | --- |
| Physician | 261 (47.3) |
| Physician and patient together | 186 (33.7) |
| Patient | 105 (19.0) |

* 152 patients had no response to this question

**Table S3. OAC uptake by patients after enrolment**

| **OAC Used** | **Frequency** | **Percent** |
| --- | --- | --- |
| **Apixaban^*^** | 27 | 45.76 |
| **Edoxaban** | 12 | 20.34 |
| **Rivaroxaban** | 10 | 16.95 |
| **VKA** | 9 | 15.25 |
| **Other OAC^*^** | 1 | 1.69 |

*Timing of OAC treatment unknown for 1 subject who received apixaban, and 1 subject who was marked as receiving ‘other OAC’ in the CRF.

**Table S4. Reason given for starting OAC treatment**

| **Reason given that OAC was started** | **N** | **Percent** |
| --- | --- | --- |
|  |  |  |
| **Stroke** | 2 | 5.6 |
| **TIA** | 1 | 2.8 |
| **Pt is no longer opposed** | 8 | 22.2 |
| **Pt no longer high bleed risk** | 7 | 19.4 |
| **Other** | 18 | 50.0 |
| **Missing** | **23** | 46.0 |

**Table S5.** **Baseline characteristics associated with those who started OAC**

| Covariate | Level | N | n (%) who started an OAC from each category |
| --- | --- | --- | --- |
| sex | Male | 414 | 33 (8.0) |
|  | Female | 290 | 26 (9.0) |
| Race grouped | Asian | 9 | 2 (22.2) |
|  | Black | 9 | 0 (0.0) |
|  | White | 601 | 49 (8.2) |
|  | Other | 41 | 6 (14.6) |
| COUNTRY | Argentina | 153 | 4 (2.6) |
|  | Brazil | 39 | 3 (7.7) |
|  | Canada | 20 | 2 (10.0) |
|  | Czech Republic | 73 | 11 (15.1) |
|  | Germany | 46 | 7 (15.2) |
|  | Hungary | 4 | 0 (0.0) |
|  | Italy | 25 | 9 (36.0) |
|  | Mexico | 44 | 4 (9.1) |
|  | Poland | 109 | 9 (8.3) |
|  | Spain | 26 | 2 (7.7) |
|  | United Kingdom | 99 | 5 (5.1) |
|  | United States of America | 66 | 3 (4.5) |
| CARESETTING | Hospital - Private | 128 | 7 (5.5) |
|  | Hospital - Public | 241 | 23 (9.5) |
|  | Office - Group | 295 | 25 (8.5) |
|  | Office - Solo | 40 | 4 (10.0) |
| Care setting Location | Hospital | 265 | 25 (9.4) |
|  | Office | 405 | 31 (7.7) |
|  | Anticoagulation clinic/thrombosis centre | 12 | 2 (16.7) |
|  | Emergency room | 4 | 1 (25.0) |
|  | Unknown | 18 | 0 (0.0) |
| Care setting Specialty | Internal medicine | 99 | 10 (10.1) |
|  | Cardiology | 482 | 41 (8.5) |
|  | Neurology | 1 | 0 (0.0) |
|  | Geriatrics | 5 | 0 (0.0) |
|  | Primary care/general practice | 112 | 7 (6.3) |
|  | Unknown | 5 | 1 (20.0) |
| Aortic disease | No Aortic Disease | 598 | 53 (8.9) |
|  | Aortic Disease | 77 | 4 (5.2) |
| Aortic Regurgitation | No Aortic Regurgitation | 666 | 56 (8.4) |
|  | Aortic Regurgitation | 24 | 2 (8.3) |
| Aortic Stenosis | No Aortic Stenosis | 655 | 57 (8.7) |
|  | Aortic Stenosis | 35 | 1 (2.9) |
| Coronary artery disease | No CAD | 469 | 40 (8.5) |
|  | CAD | 189 | 15 (7.9) |
| Carotid disease | No Carotid Disease | 611 | 50 (8.2) |
|  | Carotid Disease | 44 | 6 (13.6) |
| Heart Failure | No HF | 439 | 35 (8.0) |
|  | HF | 259 | 24 (9.3) |
| NYHA class congestive heart failure | I | 28 | 5 (17.9) |
|  | II | 156 | 13 (8.3) |
|  | III | 53 | 4 (7.5) |
|  | IV | 2 | 1 |
| Congenital cardiac anomaly | No Congenital cardiac Abnormality | 688 | 58 (8.4) |
|  | Congenital Cardiac Abnormality | 7 | 0 (0.0) |
| History of bleeding | No History of Bleed | 416 | 40 (9.6) |
|  | History of Bleed | 283 | 18 (6.4) |
| History of GI bleeding | No Hx of GI Bleed | 550 | 46 (8.4) |
|  | Hx of GI Bleed | 139 | 11 (7.9) |
| History of intracranial bleeding | No Hx of IC Bleed | 651 | 57 (8.8) |
|  | Hx of IC Bleed | 43 | 1 (2.3) |
| History of MI or unstable angina | No Hx of MI or USA | 600 | 49 (8.2) |
|  | Hx of MI or USA | 93 | 9 (9.7) |
| History of VTE | No History of VTE | 667 | 56 (8.4) |
|  | History of VTE | 26 | 3 (11.5) |
| Systemic embolization | No History of SE | 680 | 57 (8.4) |
|  | History of SE | 11 | 2 (18.2) |
| Mitral Regurgitation | No Mitral Regurgitation | 640 | 56 (8.8) |
|  | Mitral Regurgitation | 50 | 2 (4.0) |
| Mitral Stenosis | No Mitral Stenosis | 680 | 58 (8.5) |
|  | Mitral Stenosis | 10 | 0 (0.0) |
| Peripheral artery disease | No PAD | 604 | 49 (8.1) |
|  | PAD | 80 | 9 (11.3) |
| Prior stroke | No Prior Stroke | 622 | 55 (8.8) |
|  | Prior Stroke | 77 | 4 (5.2) |
| Prior transient ischaemic attack | No prior TIA | 656 | 55 (8.4) |
|  | Prior TIA | 37 | 4 (10.8) |
| Rheumatic heart disease | No Rheumatic Heart Disease | 684 | 57 (8.3) |
|  | Rheumatic Heart Disease | 7 | 0 (0.0) |
| Tricuspid Regurgitation | No Tricuspid Regurgitation | 647 | 55 (8.5) |
|  | Tricuspid Regurgitation | 43 | 3 (7.0) |
| Clinically significant valve disease | No Significant Valve Disease | 590 | 52 (8.8) |
|  | Significant Valve Disease | 100 | 6 (6.0) |
| Prior Stroke/TIA/SE | No Prior Stroke/TIA/SE | 580 | 49 (8.4) |
|  | Prior Stroke/TIA/SE | 112 | 10 (8.9) |
| Alcohol consumption | None | 482 | 33 (6.8) |
|  | Less than 8 drinks per week | 146 | 16 (11.0) |
|  | Greater or = 8 drinks per week | 19 | 0 (0.0) |
|  | Great than or = 14 drinks per week | 8 | 2 (25.0) |
| Cancer with chemotherapy, systemic treatment or major surgery | No History of Cancer | 619 | 55 (8.9) |
|  | History of Cancer | 76 | 4 (5.3) |
| Cirrhosis or Bilirubin > 2x normal with AST/ALT/AP > 3x normal | No History of Cirrhosis | 654 | 56 (8.6) |
|  | History of Cirrhosis | 16 | 0 (0.0) |
| Chronic kidney disease (Stage) | I | 37 | 9 (24.3) |
|  | II | 83 | 3 (3.6) |
|  | IIIa | 58 | 4 (6.9) |
|  | IIIb | 58 | 4 (6.9) |
|  | IV | 49 | 5 (10.2) |
|  | V | 40 | 4 (10.0) |
| Hypercholesterolaemia | No History of Hypercholesterolaemia | 359 | 22 (6.1) |
|  | History of Hypercholesterolaemia | 324 | 34 (10.5) |
| Hypothyroidism | No Hypothyroidism | 581 | 49 (8.4) |
|  | Hypothyroidism | 111 | 7 (6.3) |
| cognitive impairment or dementia | No Cognitive Impairment | 652 | 58 (8.9) |
|  | Cognitive Impairment | 49 | 1 (2.0) |
| Sleep apnoea | No Sleep Apnoea | 639 | 52 (8.1) |
|  | Sleep Apnoea | 34 | 3 (8.8) |
| Smoker | Non-Smoker | 445 | 34 (7.6) |
|  | Ex-Smoker | 184 | 12 (6.5) |
|  | Current smoker | 45 | 8 (17.8) |
| Hypertension | No Hypertension | 96 | 7 (7.3) |
|  | Hypertension | 604 | 52 (8.6) |
| Diabetes | No Diabetes | 502 | 33 (6.6) |
|  | Diabetes | 196 | 25 (12.8) |
| Pattern of atrial fibrillation/flutter | Permanent | 286 | 14 (4.9) |
|  | Persistent | 72 | 11 (15.3) |
|  | Paroxysmal | 304 | 29 (9.5) |
|  | Type not yet determined | 15 | 3 (20.0) |
| Timing of AF | Incidence | 53 | 12 (22.6) |
|  | Prevalence | 650 | 47 (7.2) |
| Investigator defined Frailty | No Frailty | 280 | 30 (10.7) |
|  | Frailty | 398 | 26 (6.5) |
| Age | < 65 | 45 | 5 (11.1) |
|  | 65-74 | 158 | 13 (8.2) |
|  | 75-84 | 277 | 27 (9.7) |
|  | >=85 | 224 | 14 (6.3) |
| BMI | <18.5 | 18 | 0 (0.0) |
|  | 18.5-24.9 | 221 | 19 (8.6) |
|  | 25-29.9 | 269 | 20 (7.4) |
|  | >=30 | 196 | 20 (10.2) |
| CHA_2_DS_2_-VA | 1-2 | 52 | 5 (9.6) |
|  | 3 | 144 | 8 (5.6) |
|  | 4 | 183 | 13 (7.1) |
|  | 5 | 143 | 15 (10.5) |
|  | >=6 | 124 | 14 (11.3) |
| HAS BLED | 0-1 | 10 | 1 (2.0) |
|  | 2 | 170 | 14 (27.5) |
|  | 3 | 302 | 26 (51.0) |
|  | >=4 | 157 | 10 (19.6) |
| Creatinine Clearance (C-G) | 15-30 | 65 | 10 (15.4) |
|  | 31-50 | 122 | 7 (5.7) |
|  | <15 | 30 | 1 (3.3) |
|  | >50 | 208 | 21 (10.1) |

**Table S6. Distribution of baseline characteristics in GARDENIA: Atrial Fibrillation Strategy**

| **Treatment Strategy** | **Level** | **Did not started OAC**  **N=645** | **Started OAC**  **N=59** |
| --- | --- | --- | --- |
| Current atrial fibrillation treatment strategy | Rhythm | 180 (31.1) | 17 (36.2) |
|  | Rate | 318 (55.0) | 19 (40.4) |
|  | Both | 80 (13.8) | 11 (23.4) |
| Prior cardioversion | Prior Cardioversion | 76 (12.1) | 9 (15.3) |
| History of ablation | History of Ablation | 47 (7.4) | 3 (5.1) |

**Table S7. Baseline Medications.**

| Baseline Medication | N=701 |
| --- | --- |
| Antiplatelet Therapy | 324 (46.2) |
| Aspirin | 283 (40.4) |
| Clopidogrel | 59 (8.4) |
| Ticagrelor | 1 (0.1) |
| Dual AP therapies (DAPT) | 29 (4.1) |
| Other AP Therapies | 11 (1.6) |
| Atrial Fibrillation Treatment | 318 (45.4) |
| Class II | 161 (23.0) |
| Class IV | 40 (5.7) |
| Digoxin | 51 (7.3) |
| Other Antiarrhythmics | 113 (16.1) |
| Other Cardiovascular Medications | 601 (85.7) |
| ACE Inhibitors | 154 (22.0) |
| Aldosterone Blockade | 94 (13.4) |
| Angiotensin Receptor Blocker | 168 (24.0) |
| Angiotensin Receptor Neprilysin Inhibitor | 18 (2.6) |
| Antiplatelets | 134 (19.1) |
| Beta Blockers | 396 (56.5) |
| Loop or other Diuretics | 243 (34.7) |
| Nitrates | 20 (2.9) |
| Oral Antidiabetic drugs | 98 (14.0) |
| SGLT-2 Inhibitors | 47 (6.7) |
| Statins | 283 (40.4) |
| Other lipid lowering drugs | 23 (3.3) |
| Alpha-blocker | 34 (4.9) |
| Non-Cardiovascular Medications | 296 (42.2) |
| Hormone Replacement Therapy | 58 (8.3) |
| Protein Pump Inhibitor | 204 (29.1) |
| Anti-retroviral agents | 1 (0.1) |
| Erythropoietin Stimulating Agents | 11 (1.6) |
| Antimycotics | 1 (0.1) |
| NSAIDS | 82 (11.7) |

**Table S8. Cause of Death**

| n (%) | Number of Deaths | |
| --- | --- | --- |
| Primary Cause of Death | 4 Months  (n=44) | 8 Month  (n=53) |
| Cardiovascular | 12 (27.3) | 14 (26.4) |
| Congestive Heart Failure | 6 (50.0) | 7 (50.0) |
| Dysrhythmia | 1 (8.3) | 1 (7.1) |
| Intracranial/spinal haemorrhage | 1 (8.3) | 1 (7.1) |
| Myocardial infarction | 1 (8.3) | 2 (14.3) |
| Sudden or Unwitnessed | 1 (8.3) | 1 (7.1) |
| Other Cardiovascular | 2 (16.7) | 2 (14.3) |
| Ischaemic stroke | 0 (0) | 0 (0) |
|  |  |  |
| Non-Cardiovascular | 21 (47.7) | 24 (45.3) |
| Accidental/trauma | 1 (4.8) | 1 (4.2) |
| Infection | 5 (23.8) | 6 (25.0) |
| Malignancy | 1 (4.8) | 1 (4.2) |
| Renal | 1 (4.8) | 2 (8.3) |
| Respiratory Failure | 2 (9.5) | 2 (8.3) |
| Sepsis | 6 (28.6) | 7 (29.2) |
| Unknown but non-CV | 1 (4.8) | 1 (4.2) |
| Other | 4 (19.0) | 4 (16.7) |
| Unknown | 11 (25.0) | 15 (28.3) |

**Table S9. ISTH Bleeding rates while not on an OAC. Patients who start an OAC are censored at the time of the OAC**

|  | K-M 4-month | | 100 Person Years (whole period) | |
| --- | --- | --- | --- | --- |
| Outcome | Events | Rate (95% CI) | Events | Rate (95% CI) |
| Major | 9 | 1.45 (0.76, 2.77) | 9 | 2.06 (1.07, 3.97) |
| Intracranial | 1 | 0.17 (0.02, 1.19) | 1 | 0.23 (0.03, 1.62) |
| Non-IC Major | 8 | 1.28 (0.64, 2.55) | 8 | 1.83 (0.92, 3.67) |
| CRNM | 2 | 0.30 (0.08, 1.21) | 2 | 0.46 (0.11, 1.83) |
| Minor | 11 | 1.79 (0.99, 3.21) | 11 | 2.54 (1.41, 4.59) |
| Major or CRNM | 11 | 1.75 (0.97, 3.15) | 11 | 2.53 (1.40, 4.57) |
| Any | 22 | 3.54 (2.34, 5.33) | 22 | 5.12 (3.37, 7.78) |

**Table S10. Clinical events based on main reason for not being treated with anticoagulants (per 100-person-year rates for end of observation time)**

| **Main reason anticoagulant was not used** | **All Cause Mortality (rate)** | **Stroke (rate)** | **Major or NCRM Bleed (rate)** |
| --- | --- | --- | --- |
| Underlying condition associated with bleeding risk | 16/109 (26.8) | 1/109 (1.6) | 3/109 (4.9) |
| Previous bleeding needing hospitalization or medical intervention | 12/119 (17.0) | 0/119 (0) | 5/119 (7.3) |
| Patient refusal to take anticoagulants | 4/112 (4.9) | 1/112 (1.2) | 0/112 (0) |
| Frailty | 2/35 (9.9) | 0/35 (0) | 1/35 (5.0) |
| Fall risk / History of traumatic falls | 4/39 (16.8) | 0/39 (0) | 0/39 (0) |
| Already taking anti-platelet drugs for other medical condition | 0/17 (0) | 0/17 (0) | 0/17 (0) |
| Previous minor or nuisance bleeding | 3/33 (12.9) | 1/33 (4.4) | 0/33 (0) |
| Chronic NSAID use (>3 times per week) | 0/14 (0) | 0/14 (0) | 0/14 (0) |
| Severe renal impairment | 3/18 (26.8) | 0/18 (0) | 1/18 (9.1) |
| Predominantly in sinus rhythm / Low AF burden | 0/27 (0) | 1/27 (4.9) | 0/27 (0) |
| Haemodialysis | 2/5 (98.2) | 0/5 (0) | 0/5 (0) |
| Anticoagulant compliance concern / Poor access to monitoring | 0/2 (0) | 0/2 (0) | 0/2 (0) |
| Cost | 0/0 | 0/0 | 0/0 |
| Cognitive Impairment | 0/0 | 0/0 | 0/0 |
| Drug interactions | 0/6 (0) | 0/6 (0) | 0/6 (0) |
| Liver disease | 0/2 (0) | 0/2 (0) | 1/2 (70.7) |
| Alcohol abuse | 0/2 (0) | 0/2 (0) | 0/2 (0) |
| Other | 5/43 (16.8) | 0/43 (0) | 0/43 (0) |
| Unknown | 12/55 (11.4) | 4/55 (3.9) | 1/55 (1.0) |

**Note:** There were 63 patients who died with an overall 100 person-year rate of 13.2 (10.3, 16.9); 8 patients with a stroke with an overall rate of 1.7 (0.8, 3.4); 12 patients with a major or clinically relevant non-major bleed with an overall rate of 2.5 (1.4, 4.5).

**Table S11. Disposition status at different time points during follow-up (697 with end of study information)**

| Disposition status | 4 Months, n (%) | 8 Months, n (%) | **End of Study,**  **n (%)** |
| --- | --- | --- | --- |
| Early withdrawal | 16 (2.3%) | 31 (4.5%) | 40 (5.7%) |
| Death | 44 (6.3%) | 53 (7.6%) | 63 (9.0%) |

**Table S12. Reasons for withdrawal**

| Reason for Withdrawal | N (%) |
| --- | --- |
| Lost to Follow-up | 9 (22.5%) |
| Withdrew Consent | 9 (22.5%) |
| Enrolled in an OAC Study | 22 (55.0%) |

**Table S13. Comparing the GARDENIA patients to the GARFIELD-AF patients with CHA_2_DS_2_-VA of 2 or greater who did not receive any OAC as their initial treatment. GARFIELD-AF is patients with newly diagnosed AF.**

| **Covariate** | **Level** | **GARDENIA**  **n=704** | **GARFIELD-AF N=12137** |
| --- | --- | --- | --- |
| Sex | Female | 290 (41.2) | 5644 (46.5) |
|  |  |  |  |
| Race/Ethnicity | Caucasian | 601 (91.1) | 6639 (56.3) |
|  | Asian | 9 (1.4) | 4052 (34.3) |
|  | Afro-Caribbean/Mixed/Other | 50 (7.6) | 1108 (9.4) |
| Type of AF | Permanent | 286 (42.2) | 1371 (11.3) |
|  | Persistent | 72 (10.6) | 1316 (10.8) |
|  | Paroxysmal | 304 (44.9) | 3403 (28.0) |
|  | New (not determined) | 15 (2.2) | 6047 (49.8) |
| Care setting location | Hospital | 265 (37.6) | 7639 (62.9) |
|  | Office/AC clinic/Thrombosis centre | 417 (59.2) | 3206 (26.4) |
|  | Emergency room | 4 (0.6) | 1292 (10.6) |
| Heart failure |  | 259 (37.1) | 3437 (28.3) |
| Acute coronary syndrome |  | 93 (13.4) | 1960 (16.2) |
| Carotid occlusive disease |  | 44 (6.7) | 389 (3.2) |
| History of bleeding |  | 283 (40.5) | 578 (4.8) |
| Hypertension |  | 604 (86.3) | 9863 (81.3) |
| Hypercholesterolemia |  | 324 (47.4) | 4782 (40.8) |
| Diabetes |  | 196 (28.1) | 3138 (25.9) |
| Cirrhosis |  | 16 (2.4) | 93 (0.8) |
| Moderate to severe CKD |  | 205 (30.7) | 1394 (12.0) |
| Dementia |  | 49 (7.0) | 289 (2.4) |
| Heavy alcohol user |  | 8 (1.2) | 226 (2.2) |
| Current smoker |  | 45 (6.7) | 1045 (9.5) |
| Age at diagnosis |  | 79.0 (73.0;86.0) | 73.0 (66.0;80.0) |
| BMI (kg/m^2^) |  | 27.0 (24.0;30.4) | 26.4 (23.5;30.1) |
| Systolic blood pressure (mmHg) |  | 129.0 (117.0;140.0) | 132.0 (120.0;145.0) |
| Diastolic blood pressure (mmHg) |  | 73.0 (67.0;80.0) | 80.0 (70.0;87.0) |
| Pulse (bpm) |  | 70.0 (62.0;80.0) | 82.0 (70.0;102.0) |
| CHA2DS2-VASc score |  | 4.0 (3.0;5.0) | 4.0 (3.0;5.0) |

**Table S14. GARFIELD estimated rates of outcomes**

| **GARFIELD Estimated Risk Rates** | **2-year rate (95% CI)** |
| --- | --- |
| ***Stroke/ SE*** |  |
| OAC Patients | 4.52 (3.75, 5.28) |
| No OAC Patients | 5.05 (4.71, 5.38) |
| ***Major bleeding*** |  |
| OAC Patients | 2.90 (2.32, 3.47) |
| No OAC Patients | 3.25 (3.05, 3.45) |

Note : The estimated rates provided assume the patients were not given an OAC for stroke prevention

**Figure S1. Cumulative incidence of OAC uptake in those patients who went on to take an OAC during the follow-up period**


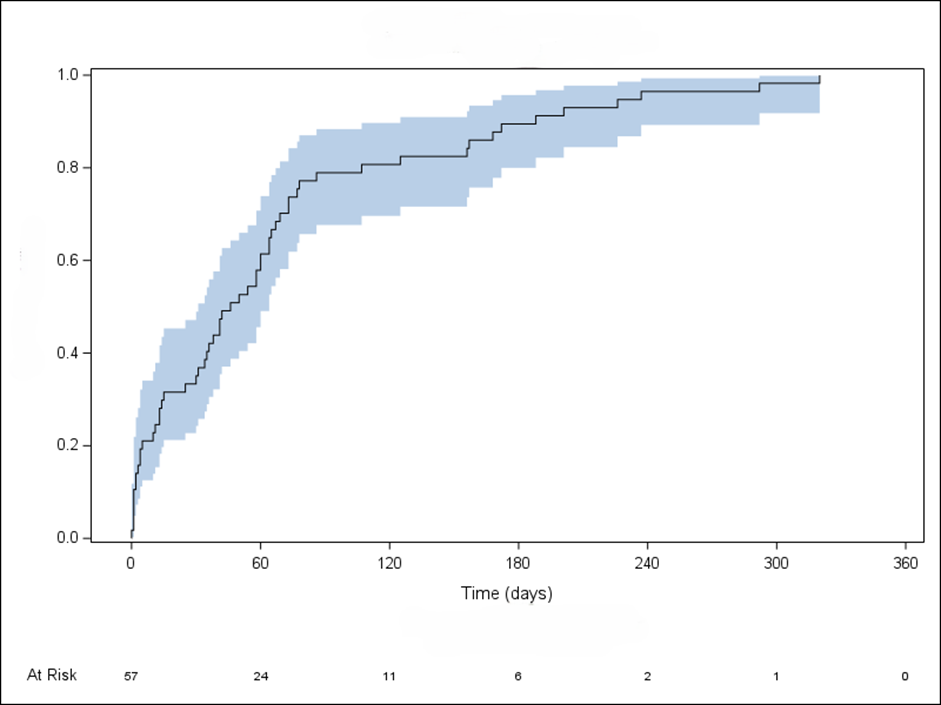


Note: Of the 697 patients with follow-up information, 57 were subsequently started on an OAC. (59 patients have an observation of OAC use but two do not have a start date for the OAC so we cannot confirm if it was before or after the start of enrolment). The median time to the start of an OAC was 42 days (11, 73).
