# Supplementary material for: Contemporary patients with atrial fibrillation are not anticoagulated despite risks of stroke - Insights from GARDENIA
Source: PLoS One. 2026 Jul 28;21(7):e0354382. doi: 10.1371/journal.pone.0354382 (PMC13411893; doi:10.1371/journal.pone.0354382)
Supplement: S1 Table — (DOCX) [file pone.0354382.s002.docx]

**Table S1. Inclusion and exclusion criteria**

| Inclusion criteria | 1. Written informed consent from the patient, or legally authorized representative, must be obtained before any assessment is performed 2. Patients with diagnosed AF or atrial flutter (documented on an electrocardiogram or monitor recording) 3. CHA₂DS₂-VASc score ≥2 (excluding female as a factor) 4. Judged by the investigator to be at increased risk of bleeding because of at least 1 of the following:    1. Age ≥70 on the day of informed consent    2. Reduced renal function (creatinine clearance <30 mL/min by Cockcroft-Gault)    3. Chronic use of NSAIDs or antiplatelet agents    4. Any other condition associated with increased risk such as a prior history of major or clinically relevant nonmajor bleeding, increased fall risk, or frailty. 5. Patients who are not treated with oral anticoagulants (only applies to Phase 1) |
| --- | --- |
| Exclusion criteria | 1. Mechanical heart valve or valve disease that is expected to require valve replacement intervention (surgical or invasive) during the course of the registry study 2. AF due to a reversible cause (e.g., cardiac surgery, pulmonary embolism, untreated hyperthyroidism, ethanol use) 3. Clinically unstable or active endocarditis or endovascular infection 4. Patients with a medical condition other than AF for which chronic use of an oral anticoagulant is indicated 5. History of left atrial appendage closure or removal 6. Life expectancy <1 year at the time of enrollment as assessed by the Investigator 7. Any medical or psychiatric condition which in the judgment of the Investigator may preclude patients of complying with study requirements for the duration of the study |
