# Supplementary material for: Contemporary patients with atrial fibrillation are not anticoagulated despite risks of stroke - Insights from GARDENIA
Source: PLoS One. 2026 Jul 28;21(7):e0354382. doi: 10.1371/journal.pone.0354382 (PMC13411893; doi:10.1371/journal.pone.0354382)
Supplement: S2 Table — (DOCX) [file pone.0354382.s003.docx]

**Table S2. The person who decided that OAC would not be used.**

| **Deciding party** | **N=552** |
| --- | --- |
| Physician | 261 (47.3) |
| Physician and patient together | 186 (33.7) |
| Patient | 105 (19.0) |

* 152 patients had no response to this question
