# Supplementary material for: Contemporary patients with atrial fibrillation are not anticoagulated despite risks of stroke - Insights from GARDENIA
Source: PLoS One. 2026 Jul 28;21(7):e0354382. doi: 10.1371/journal.pone.0354382 (PMC13411893; doi:10.1371/journal.pone.0354382)
Supplement: S3 Table — (DOCX) [file pone.0354382.s004.docx]

**Table S3. OAC uptake by patients after enrolment**

| **OAC Used** | **Frequency** | **Percent** |
| --- | --- | --- |
| **Apixaban^*^** | 27 | 45.76 |
| **Edoxaban** | 12 | 20.34 |
| **Rivaroxaban** | 10 | 16.95 |
| **VKA** | 9 | 15.25 |
| **Other OAC^*^** | 1 | 1.69 |

*Timing of OAC treatment unknown for 1 subject who received apixaban, and 1 subject who was marked as receiving ‘other OAC’ in the CRF.
