# Supplementary material for: Contemporary patients with atrial fibrillation are not anticoagulated despite risks of stroke - Insights from GARDENIA
Source: PLoS One. 2026 Jul 28;21(7):e0354382. doi: 10.1371/journal.pone.0354382 (PMC13411893; doi:10.1371/journal.pone.0354382)
Supplement: S4 Table — (DOCX) [file pone.0354382.s005.docx]

**Table S4. Reason given for starting OAC treatment**

| **Reason given that OAC was started** | **N** | **Percent** |
| --- | --- | --- |
|  |  |  |
| **Stroke** | 2 | 5.6 |
| **TIA** | 1 | 2.8 |
| **Pt is no longer opposed** | 8 | 22.2 |
| **Pt no longer high bleed risk** | 7 | 19.4 |
| **Other** | 18 | 50.0 |
| **Missing** | **23** | 46.0 |
