# Supplementary material for: Contemporary patients with atrial fibrillation are not anticoagulated despite risks of stroke - Insights from GARDENIA
Source: PLoS One. 2026 Jul 28;21(7):e0354382. doi: 10.1371/journal.pone.0354382 (PMC13411893; doi:10.1371/journal.pone.0354382)
Supplement: S5 Table — (DOCX) [file pone.0354382.s006.docx]

**Table S5.** **Baseline characteristics associated with those who started OAC**

| Covariate | Level | N | n (%) who started an OAC from each category |
| --- | --- | --- | --- |
| sex | Male | 414 | 33 (8.0) |
|  | Female | 290 | 26 (9.0) |
| Race grouped | Asian | 9 | 2 (22.2) |
|  | Black | 9 | 0 (0.0) |
|  | White | 601 | 49 (8.2) |
|  | Other | 41 | 6 (14.6) |
| COUNTRY | Argentina | 153 | 4 (2.6) |
|  | Brazil | 39 | 3 (7.7) |
|  | Canada | 20 | 2 (10.0) |
|  | Czech Republic | 73 | 11 (15.1) |
|  | Germany | 46 | 7 (15.2) |
|  | Hungary | 4 | 0 (0.0) |
|  | Italy | 25 | 9 (36.0) |
|  | Mexico | 44 | 4 (9.1) |
|  | Poland | 109 | 9 (8.3) |
|  | Spain | 26 | 2 (7.7) |
|  | United Kingdom | 99 | 5 (5.1) |
|  | United States of America | 66 | 3 (4.5) |
| CARESETTING | Hospital - Private | 128 | 7 (5.5) |
|  | Hospital - Public | 241 | 23 (9.5) |
|  | Office - Group | 295 | 25 (8.5) |
|  | Office - Solo | 40 | 4 (10.0) |
| Care setting Location | Hospital | 265 | 25 (9.4) |
|  | Office | 405 | 31 (7.7) |
|  | Anticoagulation clinic/thrombosis centre | 12 | 2 (16.7) |
|  | Emergency room | 4 | 1 (25.0) |
|  | Unknown | 18 | 0 (0.0) |
| Care setting Specialty | Internal medicine | 99 | 10 (10.1) |
|  | Cardiology | 482 | 41 (8.5) |
|  | Neurology | 1 | 0 (0.0) |
|  | Geriatrics | 5 | 0 (0.0) |
|  | Primary care/general practice | 112 | 7 (6.3) |
|  | Unknown | 5 | 1 (20.0) |
| Aortic disease | No Aortic Disease | 598 | 53 (8.9) |
|  | Aortic Disease | 77 | 4 (5.2) |
| Aortic Regurgitation | No Aortic Regurgitation | 666 | 56 (8.4) |
|  | Aortic Regurgitation | 24 | 2 (8.3) |
| Aortic Stenosis | No Aortic Stenosis | 655 | 57 (8.7) |
|  | Aortic Stenosis | 35 | 1 (2.9) |
| Coronary artery disease | No CAD | 469 | 40 (8.5) |
|  | CAD | 189 | 15 (7.9) |
| Carotid disease | No Carotid Disease | 611 | 50 (8.2) |
|  | Carotid Disease | 44 | 6 (13.6) |
| Heart Failure | No HF | 439 | 35 (8.0) |
|  | HF | 259 | 24 (9.3) |
| NYHA class congestive heart failure | I | 28 | 5 (17.9) |
|  | II | 156 | 13 (8.3) |
|  | III | 53 | 4 (7.5) |
|  | IV | 2 | 1 |
| Congenital cardiac anomaly | No Congenital cardiac Abnormality | 688 | 58 (8.4) |
|  | Congenital Cardiac Abnormality | 7 | 0 (0.0) |
| History of bleeding | No History of Bleed | 416 | 40 (9.6) |
|  | History of Bleed | 283 | 18 (6.4) |
| History of GI bleeding | No Hx of GI Bleed | 550 | 46 (8.4) |
|  | Hx of GI Bleed | 139 | 11 (7.9) |
| History of intracranial bleeding | No Hx of IC Bleed | 651 | 57 (8.8) |
|  | Hx of IC Bleed | 43 | 1 (2.3) |
| History of MI or unstable angina | No Hx of MI or USA | 600 | 49 (8.2) |
|  | Hx of MI or USA | 93 | 9 (9.7) |
| History of VTE | No History of VTE | 667 | 56 (8.4) |
|  | History of VTE | 26 | 3 (11.5) |
| Systemic embolization | No History of SE | 680 | 57 (8.4) |
|  | History of SE | 11 | 2 (18.2) |
| Mitral Regurgitation | No Mitral Regurgitation | 640 | 56 (8.8) |
|  | Mitral Regurgitation | 50 | 2 (4.0) |
| Mitral Stenosis | No Mitral Stenosis | 680 | 58 (8.5) |
|  | Mitral Stenosis | 10 | 0 (0.0) |
| Peripheral artery disease | No PAD | 604 | 49 (8.1) |
|  | PAD | 80 | 9 (11.3) |
| Prior stroke | No Prior Stroke | 622 | 55 (8.8) |
|  | Prior Stroke | 77 | 4 (5.2) |
| Prior transient ischaemic attack | No prior TIA | 656 | 55 (8.4) |
|  | Prior TIA | 37 | 4 (10.8) |
| Rheumatic heart disease | No Rheumatic Heart Disease | 684 | 57 (8.3) |
|  | Rheumatic Heart Disease | 7 | 0 (0.0) |
| Tricuspid Regurgitation | No Tricuspid Regurgitation | 647 | 55 (8.5) |
|  | Tricuspid Regurgitation | 43 | 3 (7.0) |
| Clinically significant valve disease | No Significant Valve Disease | 590 | 52 (8.8) |
|  | Significant Valve Disease | 100 | 6 (6.0) |
| Prior Stroke/TIA/SE | No Prior Stroke/TIA/SE | 580 | 49 (8.4) |
|  | Prior Stroke/TIA/SE | 112 | 10 (8.9) |
| Alcohol consumption | None | 482 | 33 (6.8) |
|  | Less than 8 drinks per week | 146 | 16 (11.0) |
|  | Greater or = 8 drinks per week | 19 | 0 (0.0) |
|  | Great than or = 14 drinks per week | 8 | 2 (25.0) |
| Cancer with chemotherapy, systemic treatment or major surgery | No History of Cancer | 619 | 55 (8.9) |
|  | History of Cancer | 76 | 4 (5.3) |
| Cirrhosis or Bilirubin > 2x normal with AST/ALT/AP > 3x normal | No History of Cirrhosis | 654 | 56 (8.6) |
|  | History of Cirrhosis | 16 | 0 (0.0) |
| Chronic kidney disease (Stage) | I | 37 | 9 (24.3) |
|  | II | 83 | 3 (3.6) |
|  | IIIa | 58 | 4 (6.9) |
|  | IIIb | 58 | 4 (6.9) |
|  | IV | 49 | 5 (10.2) |
|  | V | 40 | 4 (10.0) |
| Hypercholesterolaemia | No History of Hypercholesterolaemia | 359 | 22 (6.1) |
|  | History of Hypercholesterolaemia | 324 | 34 (10.5) |
| Hypothyroidism | No Hypothyroidism | 581 | 49 (8.4) |
|  | Hypothyroidism | 111 | 7 (6.3) |
| cognitive impairment or dementia | No Cognitive Impairment | 652 | 58 (8.9) |
|  | Cognitive Impairment | 49 | 1 (2.0) |
| Sleep apnoea | No Sleep Apnoea | 639 | 52 (8.1) |
|  | Sleep Apnoea | 34 | 3 (8.8) |
| Smoker | Non-Smoker | 445 | 34 (7.6) |
|  | Ex-Smoker | 184 | 12 (6.5) |
|  | Current smoker | 45 | 8 (17.8) |
| Hypertension | No Hypertension | 96 | 7 (7.3) |
|  | Hypertension | 604 | 52 (8.6) |
| Diabetes | No Diabetes | 502 | 33 (6.6) |
|  | Diabetes | 196 | 25 (12.8) |
| Pattern of atrial fibrillation/flutter | Permanent | 286 | 14 (4.9) |
|  | Persistent | 72 | 11 (15.3) |
|  | Paroxysmal | 304 | 29 (9.5) |
|  | Type not yet determined | 15 | 3 (20.0) |
| Timing of AF | Incidence | 53 | 12 (22.6) |
|  | Prevalence | 650 | 47 (7.2) |
| Investigator defined Frailty | No Frailty | 280 | 30 (10.7) |
|  | Frailty | 398 | 26 (6.5) |
| Age | < 65 | 45 | 5 (11.1) |
|  | 65-74 | 158 | 13 (8.2) |
|  | 75-84 | 277 | 27 (9.7) |
|  | >=85 | 224 | 14 (6.3) |
| BMI | <18.5 | 18 | 0 (0.0) |
|  | 18.5-24.9 | 221 | 19 (8.6) |
|  | 25-29.9 | 269 | 20 (7.4) |
|  | >=30 | 196 | 20 (10.2) |
| CHA_2_DS_2_-VA | 1-2 | 52 | 5 (9.6) |
|  | 3 | 144 | 8 (5.6) |
|  | 4 | 183 | 13 (7.1) |
|  | 5 | 143 | 15 (10.5) |
|  | >=6 | 124 | 14 (11.3) |
| HAS BLED | 0-1 | 10 | 1 (2.0) |
|  | 2 | 170 | 14 (27.5) |
|  | 3 | 302 | 26 (51.0) |
|  | >=4 | 157 | 10 (19.6) |
| Creatinine Clearance (C-G) | 15-30 | 65 | 10 (15.4) |
|  | 31-50 | 122 | 7 (5.7) |
|  | <15 | 30 | 1 (3.3) |
|  | >50 | 208 | 21 (10.1) |
