# Supplementary material for: Contemporary patients with atrial fibrillation are not anticoagulated despite risks of stroke - Insights from GARDENIA
Source: PLoS One. 2026 Jul 28;21(7):e0354382. doi: 10.1371/journal.pone.0354382 (PMC13411893; doi:10.1371/journal.pone.0354382)
Supplement: S6 Table — (DOCX) [file pone.0354382.s007.docx]

**Table S6. Distribution of baseline characteristics in GARDENIA: Atrial Fibrillation Strategy**

| **Treatment Strategy** | **Level** | **Did not started OAC**  **N=645** | **Started OAC**  **N=59** |
| --- | --- | --- | --- |
| Current atrial fibrillation treatment strategy | Rhythm | 180 (31.1) | 17 (36.2) |
|  | Rate | 318 (55.0) | 19 (40.4) |
|  | Both | 80 (13.8) | 11 (23.4) |
| Prior cardioversion | Prior Cardioversion | 76 (12.1) | 9 (15.3) |
| History of ablation | History of Ablation | 47 (7.4) | 3 (5.1) |
