# Supplementary material for: Contemporary patients with atrial fibrillation are not anticoagulated despite risks of stroke - Insights from GARDENIA
Source: PLoS One. 2026 Jul 28;21(7):e0354382. doi: 10.1371/journal.pone.0354382 (PMC13411893; doi:10.1371/journal.pone.0354382)
Supplement: S7 Table — (DOCX) [file pone.0354382.s008.docx]

**Table S7. Baseline Medications.**

| Baseline Medication | N=701 |
| --- | --- |
| Antiplatelet Therapy | 324 (46.2) |
| Aspirin | 283 (40.4) |
| Clopidogrel | 59 (8.4) |
| Ticagrelor | 1 (0.1) |
| Dual AP therapies (DAPT) | 29 (4.1) |
| Other AP Therapies | 11 (1.6) |
| Atrial Fibrillation Treatment | 318 (45.4) |
| Class II | 161 (23.0) |
| Class IV | 40 (5.7) |
| Digoxin | 51 (7.3) |
| Other Antiarrhythmics | 113 (16.1) |
| Other Cardiovascular Medications | 601 (85.7) |
| ACE Inhibitors | 154 (22.0) |
| Aldosterone Blockade | 94 (13.4) |
| Angiotensin Receptor Blocker | 168 (24.0) |
| Angiotensin Receptor Neprilysin Inhibitor | 18 (2.6) |
| Antiplatelets | 134 (19.1) |
| Beta Blockers | 396 (56.5) |
| Loop or other Diuretics | 243 (34.7) |
| Nitrates | 20 (2.9) |
| Oral Antidiabetic drugs | 98 (14.0) |
| SGLT-2 Inhibitors | 47 (6.7) |
| Statins | 283 (40.4) |
| Other lipid lowering drugs | 23 (3.3) |
| Alpha-blocker | 34 (4.9) |
| Non-Cardiovascular Medications | 296 (42.2) |
| Hormone Replacement Therapy | 58 (8.3) |
| Protein Pump Inhibitor | 204 (29.1) |
| Anti-retroviral agents | 1 (0.1) |
| Erythropoietin Stimulating Agents | 11 (1.6) |
| Antimycotics | 1 (0.1) |
| NSAIDS | 82 (11.7) |
