# Supplementary material for: Contemporary patients with atrial fibrillation are not anticoagulated despite risks of stroke - Insights from GARDENIA
Source: PLoS One. 2026 Jul 28;21(7):e0354382. doi: 10.1371/journal.pone.0354382 (PMC13411893; doi:10.1371/journal.pone.0354382)
Supplement: S8 Table — (DOCX) [file pone.0354382.s009.docx]

**Table S8. Cause of Death**

| n (%) | Number of Deaths | |
| --- | --- | --- |
| Primary Cause of Death | 4 Months  (n=44) | 8 Month  (n=53) |
| Cardiovascular | 12 (27.3) | 14 (26.4) |
| Congestive Heart Failure | 6 (50.0) | 7 (50.0) |
| Dysrhythmia | 1 (8.3) | 1 (7.1) |
| Intracranial/spinal haemorrhage | 1 (8.3) | 1 (7.1) |
| Myocardial infarction | 1 (8.3) | 2 (14.3) |
| Sudden or Unwitnessed | 1 (8.3) | 1 (7.1) |
| Other Cardiovascular | 2 (16.7) | 2 (14.3) |
| Ischaemic stroke | 0 (0) | 0 (0) |
|  |  |  |
| Non-Cardiovascular | 21 (47.7) | 24 (45.3) |
| Accidental/trauma | 1 (4.8) | 1 (4.2) |
| Infection | 5 (23.8) | 6 (25.0) |
| Malignancy | 1 (4.8) | 1 (4.2) |
| Renal | 1 (4.8) | 2 (8.3) |
| Respiratory Failure | 2 (9.5) | 2 (8.3) |
| Sepsis | 6 (28.6) | 7 (29.2) |
| Unknown but non-CV | 1 (4.8) | 1 (4.2) |
| Other | 4 (19.0) | 4 (16.7) |
| Unknown | 11 (25.0) | 15 (28.3) |
