# Supplementary material for: Contemporary patients with atrial fibrillation are not anticoagulated despite risks of stroke - Insights from GARDENIA
Source: PLoS One. 2026 Jul 28;21(7):e0354382. doi: 10.1371/journal.pone.0354382 (PMC13411893; doi:10.1371/journal.pone.0354382)
Supplement: S9 Table — Patients who start an OAC are censored at the time of the OAC. (DOCX) [file pone.0354382.s010.docx]

**Table S9. ISTH Bleeding rates while not on an OAC. Patients who start an OAC are censored at the time of the OAC**

|  | K-M 4-month | | 100 Person Years (whole period) | |
| --- | --- | --- | --- | --- |
| Outcome | Events | Rate (95% CI) | Events | Rate (95% CI) |
| Major | 9 | 1.45 (0.76, 2.77) | 9 | 2.06 (1.07, 3.97) |
| Intracranial | 1 | 0.17 (0.02, 1.19) | 1 | 0.23 (0.03, 1.62) |
| Non-IC Major | 8 | 1.28 (0.64, 2.55) | 8 | 1.83 (0.92, 3.67) |
| CRNM | 2 | 0.30 (0.08, 1.21) | 2 | 0.46 (0.11, 1.83) |
| Minor | 11 | 1.79 (0.99, 3.21) | 11 | 2.54 (1.41, 4.59) |
| Major or CRNM | 11 | 1.75 (0.97, 3.15) | 11 | 2.53 (1.40, 4.57) |
| Any | 22 | 3.54 (2.34, 5.33) | 22 | 5.12 (3.37, 7.78) |
