# Supplementary material for: Contemporary patients with atrial fibrillation are not anticoagulated despite risks of stroke - Insights from GARDENIA
Source: PLoS One. 2026 Jul 28;21(7):e0354382. doi: 10.1371/journal.pone.0354382 (PMC13411893; doi:10.1371/journal.pone.0354382)
Supplement: S10 Table — (DOCX) [file pone.0354382.s011.docx]

**Table S10. Clinical events based on main reason for not being treated with anticoagulants (per 100-person-year rates for end of observation time)**

| **Main reason anticoagulant was not used** | **All Cause Mortality (rate)** | **Stroke (rate)** | **Major or NCRM Bleed (rate)** |
| --- | --- | --- | --- |
| Underlying condition associated with bleeding risk | 16/109 (26.8) | 1/109 (1.6) | 3/109 (4.9) |
| Previous bleeding needing hospitalization or medical intervention | 12/119 (17.0) | 0/119 (0) | 5/119 (7.3) |
| Patient refusal to take anticoagulants | 4/112 (4.9) | 1/112 (1.2) | 0/112 (0) |
| Frailty | 2/35 (9.9) | 0/35 (0) | 1/35 (5.0) |
| Fall risk / History of traumatic falls | 4/39 (16.8) | 0/39 (0) | 0/39 (0) |
| Already taking anti-platelet drugs for other medical condition | 0/17 (0) | 0/17 (0) | 0/17 (0) |
| Previous minor or nuisance bleeding | 3/33 (12.9) | 1/33 (4.4) | 0/33 (0) |
| Chronic NSAID use (>3 times per week) | 0/14 (0) | 0/14 (0) | 0/14 (0) |
| Severe renal impairment | 3/18 (26.8) | 0/18 (0) | 1/18 (9.1) |
| Predominantly in sinus rhythm / Low AF burden | 0/27 (0) | 1/27 (4.9) | 0/27 (0) |
| Haemodialysis | 2/5 (98.2) | 0/5 (0) | 0/5 (0) |
| Anticoagulant compliance concern / Poor access to monitoring | 0/2 (0) | 0/2 (0) | 0/2 (0) |
| Cost | 0/0 | 0/0 | 0/0 |
| Cognitive Impairment | 0/0 | 0/0 | 0/0 |
| Drug interactions | 0/6 (0) | 0/6 (0) | 0/6 (0) |
| Liver disease | 0/2 (0) | 0/2 (0) | 1/2 (70.7) |
| Alcohol abuse | 0/2 (0) | 0/2 (0) | 0/2 (0) |
| Other | 5/43 (16.8) | 0/43 (0) | 0/43 (0) |
| Unknown | 12/55 (11.4) | 4/55 (3.9) | 1/55 (1.0) |

**Note:** There were 63 patients who died with an overall 100 person-year rate of 13.2 (10.3, 16.9); 8 patients with a stroke with an overall rate of 1.7 (0.8, 3.4); 12 patients with a major or clinically relevant non-major bleed with an overall rate of 2.5 (1.4, 4.5).
