# Supplementary material for: Contemporary patients with atrial fibrillation are not anticoagulated despite risks of stroke - Insights from GARDENIA
Source: PLoS One. 2026 Jul 28;21(7):e0354382. doi: 10.1371/journal.pone.0354382 (PMC13411893; doi:10.1371/journal.pone.0354382)
Supplement: S11 Table — (DOCX) [file pone.0354382.s012.docx]

**Table S11. Disposition status at different time points during follow-up (697 with end of study information)**

| Disposition status | 4 Months, n (%) | 8 Months, n (%) | **End of Study,**  **n (%)** |
| --- | --- | --- | --- |
| Early withdrawal | 16 (2.3%) | 31 (4.5%) | 40 (5.7%) |
| Death | 44 (6.3%) | 53 (7.6%) | 63 (9.0%) |
