# Supplementary material for: Contemporary patients with atrial fibrillation are not anticoagulated despite risks of stroke - Insights from GARDENIA
Source: PLoS One. 2026 Jul 28;21(7):e0354382. doi: 10.1371/journal.pone.0354382 (PMC13411893; doi:10.1371/journal.pone.0354382)
Supplement: S12 Table — (DOCX) [file pone.0354382.s013.docx]

**Table S12. Reasons for withdrawal**

| Reason for Withdrawal | N (%) |
| --- | --- |
| Lost to Follow-up | 9 (22.5%) |
| Withdrew Consent | 9 (22.5%) |
| Enrolled in an OAC Study | 22 (55.0%) |
