# Supplementary material for: Contemporary patients with atrial fibrillation are not anticoagulated despite risks of stroke - Insights from GARDENIA
Source: PLoS One. 2026 Jul 28;21(7):e0354382. doi: 10.1371/journal.pone.0354382 (PMC13411893; doi:10.1371/journal.pone.0354382)
Supplement: S13 Table — GARFIELD-AF is patients with newly diagnosed AF. (DOCX) [file pone.0354382.s014.docx]

**Table S13. Comparing the GARDENIA patients to the GARFIELD-AF patients with CHA_2_DS_2_-VA of 2 or greater who did not receive any OAC as their initial treatment. GARFIELD-AF is patients with newly diagnosed AF.**

| **Covariate** | **Level** | **GARDENIA**  **n=704** | **GARFIELD-AF N=12137** |
| --- | --- | --- | --- |
| Sex | Female | 290 (41.2) | 5644 (46.5) |
|  |  |  |  |
| Race/Ethnicity | Caucasian | 601 (91.1) | 6639 (56.3) |
|  | Asian | 9 (1.4) | 4052 (34.3) |
|  | Afro-Caribbean/Mixed/Other | 50 (7.6) | 1108 (9.4) |
| Type of AF | Permanent | 286 (42.2) | 1371 (11.3) |
|  | Persistent | 72 (10.6) | 1316 (10.8) |
|  | Paroxysmal | 304 (44.9) | 3403 (28.0) |
|  | New (not determined) | 15 (2.2) | 6047 (49.8) |
| Care setting location | Hospital | 265 (37.6) | 7639 (62.9) |
|  | Office/AC clinic/Thrombosis centre | 417 (59.2) | 3206 (26.4) |
|  | Emergency room | 4 (0.6) | 1292 (10.6) |
| Heart failure |  | 259 (37.1) | 3437 (28.3) |
| Acute coronary syndrome |  | 93 (13.4) | 1960 (16.2) |
| Carotid occlusive disease |  | 44 (6.7) | 389 (3.2) |
| History of bleeding |  | 283 (40.5) | 578 (4.8) |
| Hypertension |  | 604 (86.3) | 9863 (81.3) |
| Hypercholesterolemia |  | 324 (47.4) | 4782 (40.8) |
| Diabetes |  | 196 (28.1) | 3138 (25.9) |
| Cirrhosis |  | 16 (2.4) | 93 (0.8) |
| Moderate to severe CKD |  | 205 (30.7) | 1394 (12.0) |
| Dementia |  | 49 (7.0) | 289 (2.4) |
| Heavy alcohol user |  | 8 (1.2) | 226 (2.2) |
| Current smoker |  | 45 (6.7) | 1045 (9.5) |
| Age at diagnosis |  | 79.0 (73.0;86.0) | 73.0 (66.0;80.0) |
| BMI (kg/m^2^) |  | 27.0 (24.0;30.4) | 26.4 (23.5;30.1) |
| Systolic blood pressure (mmHg) |  | 129.0 (117.0;140.0) | 132.0 (120.0;145.0) |
| Diastolic blood pressure (mmHg) |  | 73.0 (67.0;80.0) | 80.0 (70.0;87.0) |
| Pulse (bpm) |  | 70.0 (62.0;80.0) | 82.0 (70.0;102.0) |
| CHA2DS2-VASc score |  | 4.0 (3.0;5.0) | 4.0 (3.0;5.0) |
