# Supplementary material for: Contemporary patients with atrial fibrillation are not anticoagulated despite risks of stroke - Insights from GARDENIA
Source: PLoS One. 2026 Jul 28;21(7):e0354382. doi: 10.1371/journal.pone.0354382 (PMC13411893; doi:10.1371/journal.pone.0354382)
Supplement: S14 Table — (DOCX) [file pone.0354382.s015.docx]

**Table S14. GARFIELD estimated rates of outcomes**

| **GARFIELD Estimated Risk Rates** | **2-year rate (95% CI)** |
| --- | --- |
| ***Stroke/ SE*** |  |
| OAC Patients | 4.52 (3.75, 5.28) |
| No OAC Patients | 5.05 (4.71, 5.38) |
| ***Major bleeding*** |  |
| OAC Patients | 2.90 (2.32, 3.47) |
| No OAC Patients | 3.25 (3.05, 3.45) |

Note : The estimated rates provided assume the patients were not given an OAC for stroke prevention
