# Supplementary figures and images for: Contemporary patients with atrial fibrillation are not anticoagulated despite risks of stroke - Insights from GARDENIA
Source: PLoS One. 2026 Jul 28;21(7):e0354382. doi: 10.1371/journal.pone.0354382 (PMC13411893; doi:10.1371/journal.pone.0354382)

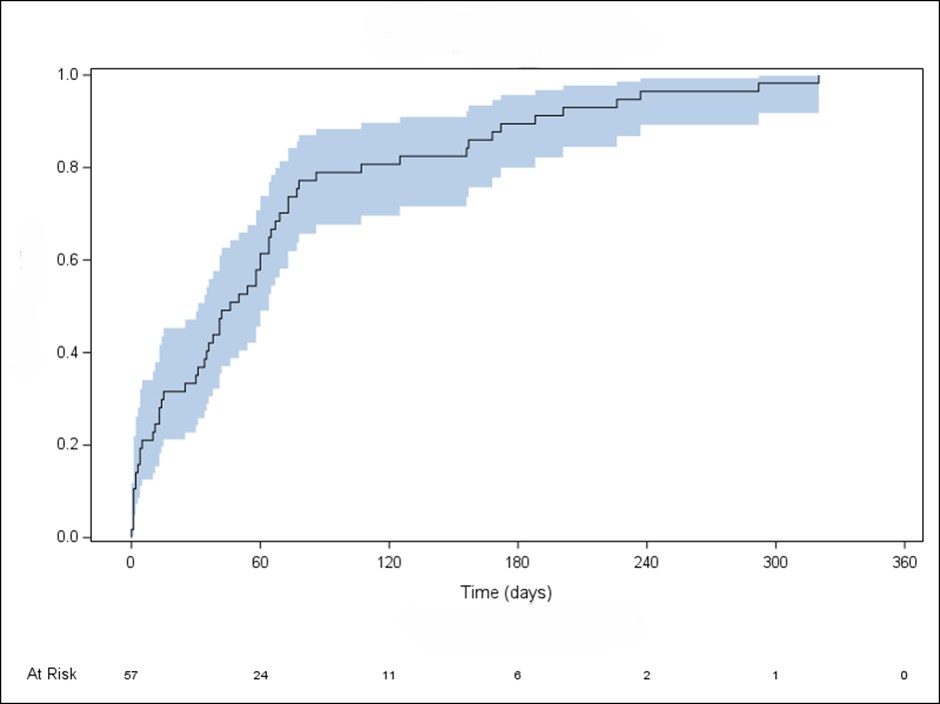

Supplement: S1 Fig — Note: Of the 697 patients with follow-up information, 57 were subsequently started on an OAC. (59 patients have an observation of OAC use but two do not have a start date for the OAC so we cannot confirm if it was before or after the start of enrolment). The median time to the start of an OAC was 42 days (11, 73). (JPG) [file pone.0354382.s016.jpg]
